# Successful Pregnancy Outcome in a Hereditary Angioedema Patient With Previous Pregnancy Losses: A Proposed Delivery Plan

**DOI:** 10.7759/cureus.54939

**Published:** 2024-02-26

**Authors:** Travis Satnarine, Alana Xavier de Almeida, Jennifer Gebbia, Gary Kleiner, Melissa Gans

**Affiliations:** 1 Pediatrics, University of Miami Miller School of Medicine, Jackson Memorial Hospital, Miami, USA; 2 Allergy and Immunology, University of Miami Miller School of Medicine, Jackson Memorial Hospital, Miami, USA

**Keywords:** delivery plan, c1 esterase inhibitor deficiency, c1 esterase inhibitor, c1 esterase, hae in pregnancy, hereditary angioedema, hae

## Abstract

This case report underscores the effective implementation of a delivery plan for a pregnant patient, focusing on a successful case study where a cesarean section, preceded by the pre-treatment of intravenous plasma-derived C1 inhibitor, resulted in the delivery of a healthy baby. The proposed delivery plan offers a systematic approach to managing hereditary angioedema during pregnancy. It recommends opting for delivery at an academic center equipped with high-risk obstetric care, obstetric anesthesia, and a level 4 Neonatal Intensive Care Unit. The plan also emphasizes the importance of early admission at the onset of labor and delineates specific protocols for both vaginal and cesarean deliveries.

## Introduction

Hereditary angioedema (HAE) is characterized by recurrent swelling of the skin and tissues caused by a deficiency in functional C1 inhibitor protein; severe cases can be life-threatening [1]. Limited treatment options and the risks of prophylactic therapy add to the difficulty of treating HAE during pregnancy. The aim of this case is to emphasize the effective control of HAE in a pregnant patient who experienced repeated early pregnancy losses, and the authors also detail a comprehensive delivery plan for pregnancy. This article was previously presented as a meeting abstract at the 2023 American College of Allergy Asthma and Immunology Annual Meeting on November 10, 2023.

## Case presentation

A 27-year-old woman with a history of monthly angioedema attacks since childhood, a father with HAE and an uncle who died from a laryngeal attack, presented for evaluation. She had a miscarriage at 12 weeks gestation and an elective first-trimester termination, both of which she attributed to abdominal angioedema attacks. The patient expressed the desire to become pregnant again.

**Figure 1:**
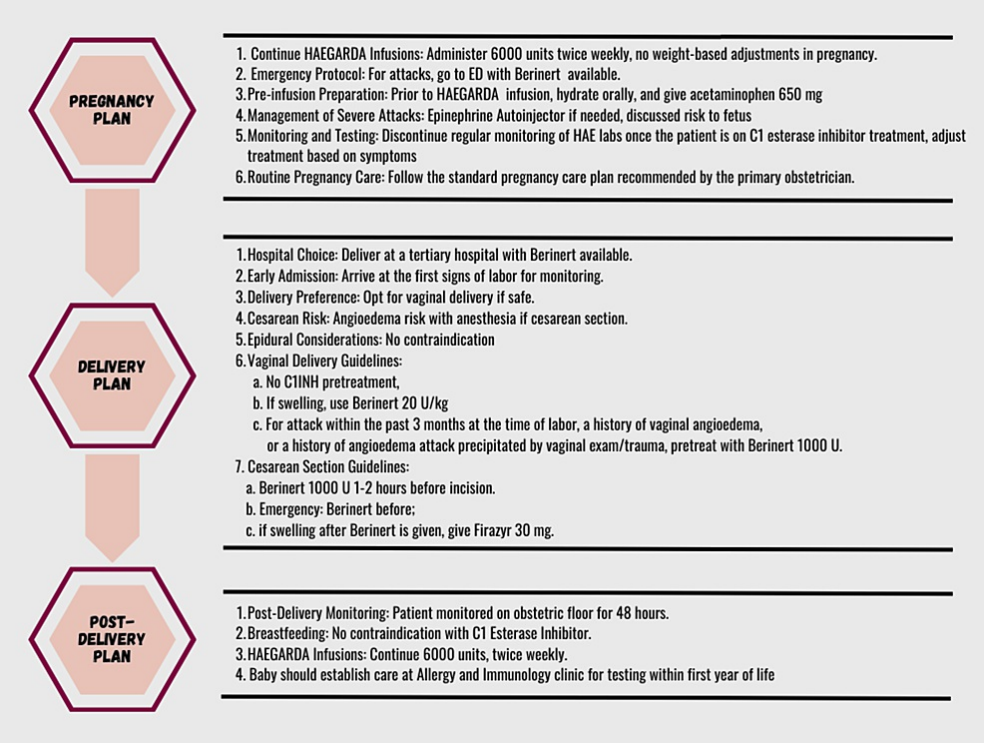
Overview of the patient's pregnancy, delivery and post-delivery plans HAEGARDA: Subcutaneous plasma-derived C1-inhibitor, ED: Emergency Department, Berinert: intravenous human plasma-derived C1-inhibitor, HAE: hereditary angioedema, C1INH: C1-inhibitor, Firazyr: bradykinin B-2 receptor antagonist (subcutaneous icatibant)

Type 2 HAE was confirmed through laboratory testing, whereby the functional activity of C1 esterase inhibitor was low at 10 mg/dL (normal range 21 - 39) and genetic testing showed the heterozygous variant c.707T>G (p.Phe236Cys) in SERPING1. She started subcutaneous (SC) plasma-derived C1-inhibitor (pdC1-INH, HAEGARDA) for prophylaxis, and bradykinin B-2 receptor antagonist, SC icatibant (Firazyr), and intravenous (IV) human pdC1-INH (Berinert) for acute attacks, see Figure 1. She was maintained on therapy for four months without any angioedema attacks before becoming pregnant. During her pregnancy, she continued regular follow-up, experiencing no angioedema attacks since starting the prophylaxis, and reported no side effects.

The patient presented for delivery at 39 weeks. Due to arrest of the active phase and inability to augment the patient further, an urgent cesarean section was performed. IV human pdC1-INH (Berinert) 1000U was administered intravenously one hour prior to the procedure and the infant was delivered via cesarean section without complications. The patient and baby were admitted to the Newborn Unit. She continued prophylactic treatment with SC pdC1-INH, which was preferred during lactation due to unknown side effects of other HAE medications on infants. She was closely monitored for 48 hours. Once no acute symptoms developed, she was discharged. The baby was confirmed postpartum to also have HAE. Approximately two months postpartum, the patient was hospitalized for treatment of acute cholecystitis. She continued to take her prophylactic medication (SC pdC1-INH). To date, she has been followed for 14 months since initiating the prophylaxis. She has had two attacks since starting prophylaxis but per the patient, very mild of short duration and significantly decreased frequently to her monthly attacks. She is now pregnant again in her second trimester with thus far a healthy pregnancy.

## Discussion

This case illustrates successful management of hereditary angioedema during pregnancy, including preventive measures. The patient suffered a miscarriage and an elective termination, which she believed were linked to HAE-related complications, and highlights the steps taken to improve future pregnancy outcomes. This emphasizes the potential link between HAE abdominal attacks and increased pregnancy loss, proposing that controlling HAE can prevent abdominal attacks and mitigate pregnancy loss.

This case presents a delivery plan that can be adopted by clinicians. This plan entails advising the patient to deliver at an academic center equipped to handle complications, with access to high-risk obstetric care, obstetric anesthesia, a level 4 Neonatal Intensive Care Unit, an ample supply of IV human pdC1-INH (Berinert) on hand, and an allergist available on call. It recommends that the patient promptly arrives at the first signs of labor, without waiting for the standard of care threshold. Vaginal delivery is preferred if safe, to minimize risks associated with angioedema attacks secondary to anesthesia during Cesarean sections. Epidural use is not typically contraindicated, and pre-treatment with pdC1-INH is not necessary for a vaginal delivery unless the patient has had an angioedema attack within the past three months at the time of labor, a history of vaginal angioedema, or a history of angioedema attack precipitated by vaginal exam or trauma. In the event of a Cesarean section, pdC1-INH 1000U would need to be administered within one to two hours of incision or immediately prior if it is an emergency. If swelling occurs after pdC1-INH, SC icatibant (Firazyr) 30mg would need to be given. Post-delivery, the patient should receive hospital monitoring for at least 48 hours.

The existing literature on the association between HAE and pregnancy loss is limited and offers conflicting findings. Bouillet et al. (2008) reported a spontaneous abortion rate of 12% among the 150 women with HAE in their study, which aligned with the 10% to 15% range observed in the general French population [1]. Martinez-Saguer et al. (2010) did not observe any instances of spontaneous abortions in their study of 22 women with HAE and 25 associated pregnancies [2]. Czaller et al. (2010) examined 118 pregnancies, where 84 neonates were delivered, and the proportion of 36 spontaneous abortions among HAE patients, similar to the general population in Hungary [3]. Gabriel et al. (2022) studied 26 women with 37 pregnancies and reported a rate comparable to that of women without HAE, with eight cases of spontaneous abortion [4]. Karabiber et al. (2022) investigated 88 pregnancies in women with HAE, resulting in 72 healthy babies but noted 12 spontaneous abortions, three stillbirths, and one neonatal death [5].

Prophylactic medication options for HAE include IV plasma-derived C1-INH such as Cinryze and SC plasma-derived C1-INH like HAEGARDA (Table 1). Both are considered safe in pregnancy according to international guidelines, expert opinions, review articles, and clinical trials. However, SC monoclonal antibody TAKHZYRO (lanadelumab-flyo) is not recommended due to insufficient data, and attenuated androgens danazol and stanozolol are contraindicated as they may cause virilization. Berotralstat (Orladeyo) is also not recommended due to insufficient data, and antifibrinolytic tranexamic acid should only be used when C1-INH concentrates are unavailable, as it can cross the placenta. On-demand treatments include IV human plasma-derived C1-INH (e.g., Berinert), recognized as safe in pregnancy, and IV recombinant C1-INH (Ruconest), which can be considered (Table 2). However, a plasma kallikrein inhibitor, SC ecallantide (Kalbitor), is not recommended due to insufficient data. Additionally, the bradykinin B-2 receptor antagonist, SC icatibant (Firazyr), has mixed data, with reports of safe administration in pregnancy noted by international guidelines, while a review article suggests refraining from recommending it until more evidence is available [1].

**Table 1:** Prophylactic Treatments for HAE in Pregnancy IV: intravenous, C1-INH: C1-inhibitor, SC: subcutaneous, HAE: hereditary angioedema

Generic Name	Example	Data
IV plasma-derived C1-INH	Cinryze	Plasma-derived C1 inhibitor concentrates are recognized as safe in pregnancy by an international guideline [6], a case report [7], expert opinion [8], review articles [9-11], clinical trial [12].
SC plasma-derived C1-INH	HAEGARDA	
SC monoclonal antibody	TAKHZYRO [lanadelumab-flyo]	Not recommended due to insufficient data [6].
Attenuated androgens	danazol and stanozolol	Contraindicated, can cause virilization [9].
Berotralstat	Orladeyo	Not recommended, insufficient data [6,9].
Antifibrinolytics	tranexamic acid	Should be used only when C1-INH concentrates are unavailable, as it can cross the placenta [9].

**Table 2:** On-Demand Treatments for HAE in Pregnancy IV: intravenous, C1-INH: C1-inhibitor, SC: subcutaneous, HAE: hereditary angioedema

Generic Name	Example	Data
IV human plasma-derived C1-INH	Berinert	Plasma-derived C1 inhibitor concentrates are recognized as safe in pregnancy by an international guideline [6], a case report [7], expert opinion [8], review articles [9-11], clinical trial [12].
IV recombinant C1-INH	Ruconest	Can be considered [9].
a plasma kallikrein inhibitor, SC ecallantide	Kalbitor	Not recommended, insufficient data [6,9].
bradykinin B-2 receptor antagonist, SC icatibant	Firazyr	There is mixed data. International guideline notes that there are reports of safe administration in pregnancy [6,13]. However, a review article states that it cannot be recommended due to need for more evidence [9].

## Conclusions

This case showcases successful HAE management during pregnancy, with a comprehensive delivery plan addressing potential complications. The insights gained emphasize the link between HAE abdominal attacks and pregnancy loss. The provided delivery plan offers practical guidance for clinicians, underlining the importance of specialized care. The insights gained from this case can be valuable for other HAE patients, as it addresses the lack of specific guidelines for delivery plans.
